# SLE-associated intestinal pseudo-obstruction: a case report and analysis of 43 cases

**DOI:** 10.3389/fimmu.2026.1756156

**Published:** 2026-04-20

**Authors:** Ting Long, Ji Li, Sheng-Guang Li, Jing Zhang, Lina Zhang, Yadan Zou, Ruohan Yu, Yanfeng Zhang

**Affiliations:** Department of Rheumatology and Immunology, Peking University International Hospital, Beijing, China

**Keywords:** immunosuppressive therapy, intestinal pseudo-obstruction, intravenous immunoglobulin, pulmonary arterial hypertension, systemic lupus erythematosus

## Abstract

**Introduction:**

Intestinal pseudo-obstruction (IPO) is a rare but serious gastrointestinal complication of systemic lupus erythematosus (SLE), often mimicking surgical obstruction. It may be reversible with prompt immunosuppressive therapy but is frequently misdiagnosed.

**Methods:**

We report a patient with SLE-IPO and a history of pulmonary arterial hypertension (PAH), a combination rarely described. A systematic review was conducted via PubMed and EMBASE through March 31, 2025. After deduplication and screening, 30 publications reporting 42 individual cases were included. Combined with our case, 43 cases were analyzed. Clinical characteristics, treatments, and outcomes were extracted and compared by publication date (≤2010 vs. >2010).

**Results:**

Among the 43 patients, 93.02% were female, with a median age of 32.00 years. IPO was the initial SLE presentation in 62.79%, and more than 90.00% occurred during active disease. Genitourinary involvement was present in 46.51%. All patients received corticosteroids. Cyclophosphamide was used in 37.21%, IVIG in 18.60%, MMF in 16.28%, and rituximab in 4.65%. After 2010, IVIG use increased (32.00% vs. 0.00%), along with broader use of MMF and biologics. IPO relapsed in 39.53%; mortality was low (2.33%). Our case achieved remission after escalation to intravenous methylprednisolone and IVIG, with MMF introduced only after bowel function recovered.

**Conclusion:**

SLE-IPO is a potentially reversible complication that often coexists with genitourinary dysmotility and only rarely with pulmonary hypertension. Timely diagnosis and aggressive immunosuppression are important to avoid irreversible damage or surgery. Evolving treatment strategies may be associated with improved outcomes, although causal inference is limited by case-level evidence.

## Introduction

Systemic lupus erythematosus (SLE) is a multisystem autoimmune disease with diverse clinical manifestations. While gastrointestinal (GI) involvement is not uncommon in SLE (1), intestinal pseudo-obstruction (IPO) is a rare but important complication characterized by impaired bowel motility without mechanical obstruction (2, 3). The pathogenesis is thought to involve autoimmune-mediated visceral myopathy and dysautonomia, often affecting the genitourinary tract in parallel (4–6). Clinically, SLE-IPO mimics acute surgical abdomen and is frequently misdiagnosed, leading to unnecessary operations and delayed immunosuppressive therapy (7).

Since the late 1990s, approximately 50 individual cases of SLE-IPO have been reported in the English-language case-report literature, and awareness has grown through case reports and small series, particularly from East Asia. More recently, two large single-center cohorts—one from Peking Union Medical College Hospital (85 cases) and another from Nanfang Hospital (32 cases)—have expanded our understanding of the clinical spectrum, radiologic features, and treatment outcomes of SLE-IPO (8, 9). However, these studies primarily focused on institutional data, with limited synthesis of global case-level details or temporal trends in management strategies.

In this case-based review, we first present a patient with SLE-IPO and a history of pulmonary arterial hypertension—a rarely reported combination. We then analyze 42 published English-language cases from 1997 to 2024, compiling a total of 43 patients. By comparing cases reported before and after 2010, we highlight evolving patterns in immunosuppressive treatment, particularly the increasing use of intravenous immunoglobulin (IVIG), mycophenolate mofetil (MMF), and rituximab in more recent years. This review aims to provide a focused clinical synthesis to support earlier recognition and optimize individualized therapy for this underrecognized but reversible complication of SLE.

## Case presentation

Patient history: A 42-year-old woman with a 15-year history of SLE presented with acute abdominal distension and vomiting. She had been diagnosed with SLE in 2009 after symptoms of intermittent fever, Raynaud’s phenomenon, and exertional dyspnea. Historical records documented severe echocardiographic pulmonary hypertension (estimated pulmonary artery pressure approximately 90 mmHg) without interstitial lung disease on chest CT. Outside records did not document chronic thromboembolic disease, and right-heart catheterization was not performed at that time. Detailed historical antiphospholipid antibody data and cardiac functional parameters beyond the echocardiographic pressure estimate were not retrievable. She was treated with high-dose glucocorticoids and monthly IV cyclophosphamide pulses; no PAH-targeted vasodilator therapy was recorded. Within three months, pulmonary pressures normalized on follow-up echocardiography. She remained on maintenance therapy with prednisone 10 mg daily and hydroxychloroquine 200 mg twice daily. In 2019, hydroxychloroquine was discontinued because of retinal toxicity (maculopathy), and azathioprine 50 mg daily was started for maintenance along with low-dose prednisone (tapered to 5 mg daily). The patient’s lupus had been clinically stable for several years on this regimen. In May 2024, routine laboratory tests indicated a mild lupus flare (white blood cell count 3×10^9/L, hemoglobin 10.5 g/dL, elevated IgG, and low complement C3/C4). Prednisone was increased to 20 mg daily (azathioprine continued), with good control. By late 2024, she had again tapered herself to prednisone 10 mg daily.

**Figure 1:**
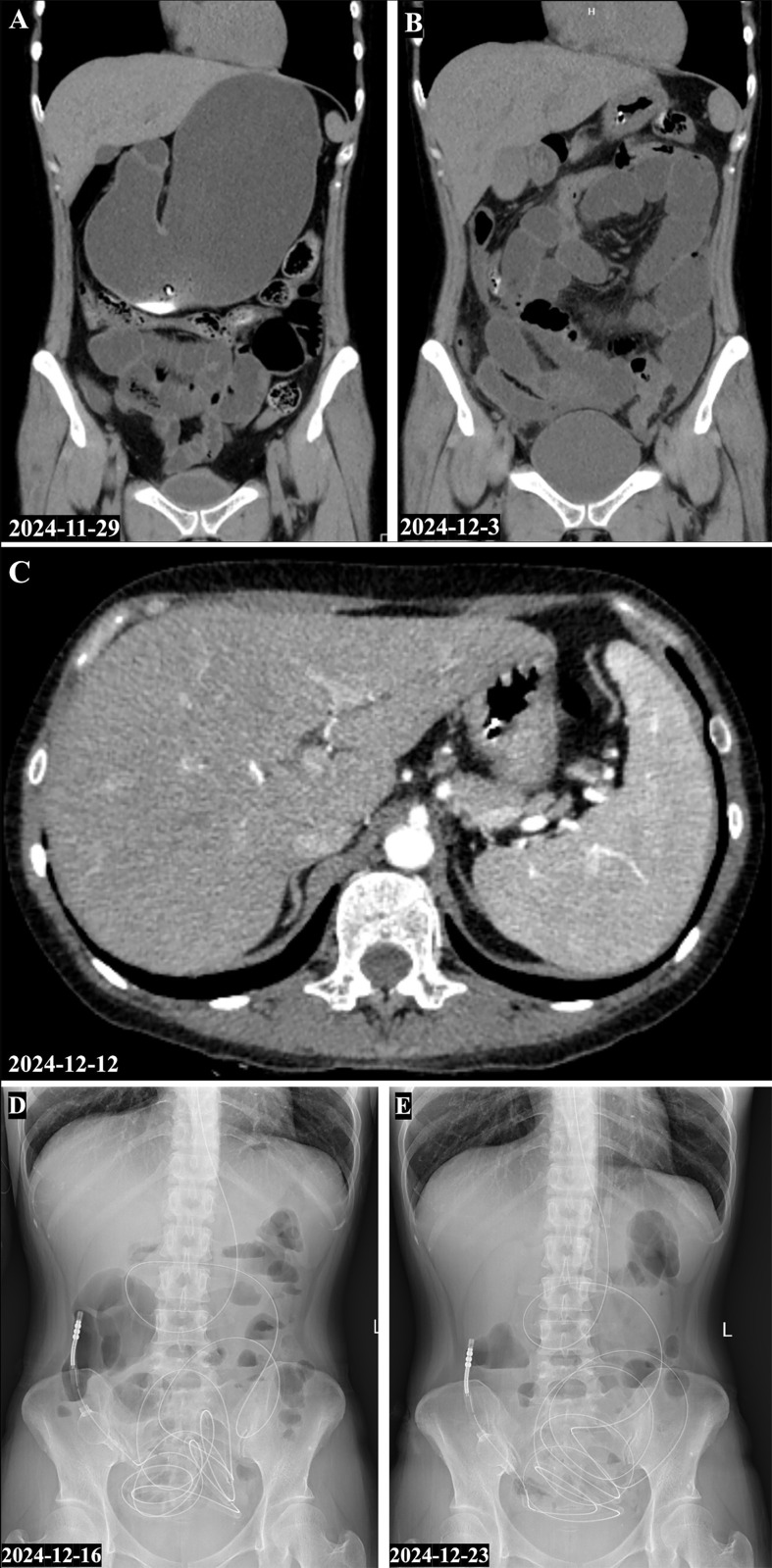
Serial imaging showing evolution and partial treatment response of intestinal pseudo-obstruction in systemic lupus erythematosus. **(A)** Abdominal and pelvic CT performed in the emergency department on November 29, 2024, showing massive gastric dilatation and intestinal distension. **(B)** Repeat CT on December 3, 2024, in the gastrointestinal surgery unit revealed progression of small-bowel dilatation. **(C)** Contrast-enhanced abdominal CT on December 12, 2024, demonstrated diffuse esophageal dilatation. **(D, E)**. Upright abdominal plain films from December 16 and December 23, 2024, following intravenous methylprednisolone 40 mg/day, continued to show prominent air-fluid levels and intestinal gas accumulation without significant improvement.

GI presentation: On November 28, 2024, the patient developed sudden epigastric fullness and intermittent abdominal pain, followed by nausea and bilious vomiting (containing coffee-ground material). The abdominal pain became diffuse and distending in quality. She had no flatus or bowel movement that day and developed fever up to 38.5 °C. She stopped taking all medications and went to the emergency department. Initial evaluation included imaging: abdominal X-ray and CT scan revealed massively dilated stomach and intestinal loops with air–fluid levels, but no mechanical obstruction (**Figure 1A**). The working diagnosis was acute gastric retention and intestinal obstruction (ileus) with probable secondary peritonitis. She was admitted to the gastroenterology service, where upper endoscopy showed retained fluid and foam in the stomach and diffuse gastric mucosal erythema and edema. A nasogastric/nasoenteric decompression tube was placed for drainage, and broad-spectrum antibiotics and supportive care (bowel rest, IV fluids and nutrition) were initiated. Despite several days of gastric decompression and medical management, her abdominal distension and pain did not significantly improve. A repeat CT on December 3, 2024, showed persistent small bowel dilation, now more pronounced and involving more distal loops (**Figure 1B**). Meanwhile, her leukopenia worsened (WBC dropped to 2.0–3.0×10^9/L). Suspecting an underlying motility disorder rather than a surgical obstruction, the team transferred the patient to the rheumatology department for further evaluation of a possible lupus-related GI manifestation.

Evaluation in rheumatology: On admission to rheumatology, the patient was ill-appearing with a distended but soft abdomen. Bowel sounds were hypoactive. Key laboratory results included: WBC 2.99×10^9/L (with neutrophils 2.09×10^9/L, lymphocytes 0.73×10^9/L), hemoglobin 9.4 g/dL, and platelets 188×10^9/L. Inflammatory markers were elevated (erythrocyte sedimentation rate 107 mm/h, C-reactive protein 8 mg/L). Comprehensive metabolic panel was unremarkable (no renal or hepatic failure). Pancreatic enzymes were mildly elevated (amylase 183 U/L, lipase 359 U/L). Stool tests were positive for occult blood. Autoimmune serologies demonstrated high-titer ANA (1:640, speckled pattern) and strongly positive anti-U1 RNP, anti-SSA/Ro60, and anti-SSA/Ro52 antibodies. Anti-dsDNA was markedly elevated at 175.8 IU/mL (normal <20). Complement C4 was low at 0.18 g/L (C3 was 1.13 g/L, within normal range). At relapse, the antiphospholipid profile showed a positive lupus anticoagulant test (dRVVT ratio 2.27), whereas anticardiolipin IgG/IgM and anti-β2-glycoprotein I IgG/IgM antibodies were negative. Infectious work-up was negative, including stool cultures, cytomegalovirus PCR, and tuberculosis screening. Upper gastrointestinal endoscopy performed during the initial surgical admission showed retained fluid and diffuse gastric mucosal erythema and edema; no tissue biopsy was obtained. A colonoscopy found no intraluminal lesions or colitis, and mucosal biopsy was not performed because the examination was macroscopically normal. A contrast-enhanced chest/abdomen/pelvis CT showed striking diffuse esophageal dilation in addition to gastric and intestinal distension (suggesting a pan-GI motility disorder), mild ascites, multiple enlarged lymph nodes, and splenomegaly (**Figure 1C**). Although this scan was not a dedicated CT angiography protocol, it showed no mesenteric arterial or venous filling defect, bowel wall ischemia, or other macrovascular occlusive pathology. These findings, in the context of active serological lupus markers and prior history, strongly pointed to intestinal pseudo-obstruction secondary to SLE rather than a mechanical obstruction. The concurrent esophageal dysmotility and ascites reinforced the likelihood of lupus-related visceral smooth-muscle involvement. Importantly, her prior severe PAH was noted as another manifestation of lupus vasculopathy; however, at this admission her cardiac ultrasound showed normal pulmonary artery pressures (the PAH remained in remission).

Treatment and course: The patient was initially started on moderate-dose corticosteroids (IV methylprednisolone 40 mg daily) along with prokinetic agents and rectal enemas, while maintaining nasogastric decompression and parenteral nutrition. However, over the next 10–14 days there was minimal improvement; serial abdominal radiographs continued to show multiple air-fluid levels and distended bowel (**Figures 1D, E**). Having ruled out infection and macrovascular thrombosis, we escalated immunosuppressive therapy to intravenous methylprednisolone 200 mg daily plus IV immunoglobulin (IVIG) 20 g daily for 5 days, given reports of IVIG benefiting SLE-IPO (6, 10, 11). Supportive measures (bowel decompression, electrolyte correction, and broad prophylactic antibiotics) were continued. The response was dramatic: within a few days of intensified immunotherapy, the patient’s abdominal pain and distension markedly diminished. She gradually resumed passing flatus and bowel movements. The nasoenteric tube was removed as her GI function improved, and she was able to advance to oral liquids and then a soft diet without recurrence of vomiting. By hospital day 20, she was tolerating a regular diet, and her abdominal examination was normal.

The patient was discharged in late December 2024 on a tapering course of oral methylprednisolone. To maintain remission and spare cyclophosphamide, we initiated mycophenolate mofetil (MMF) 1.5 g/day only after bowel function had recovered and oral intake had been re-established, rather than during the initial obstructive phase. At a 3-month follow-up (March 2025), she was doing well: methylprednisolone had been tapered to 16 mg daily, and she had experienced no further GI symptoms (normal appetite, no bloating or vomiting, regular bowel movements). Her SLE was quiescent with stable blood counts, improving complement levels, and no active PAH. This outcome underscores that prompt high-dose immunosuppressive therapy can reverse even severe SLE-related IPO.

## Literature review methods

We conducted a systematic literature search in PubMed and EMBASE through March 31, 2025, using combinations of the keywords “systemic lupus erythematosus,” “intestinal pseudo-obstruction,” “pseudo-obstruction,” and “ileus.” Articles were screened independently by two reviewers. Eligible studies included case reports or case series describing patients diagnosed with SLE who developed intestinal pseudo-obstruction based on clinical and imaging findings, with no evidence of mechanical obstruction. Because the term “lupus enteritis” is often used for vasculitic bowel involvement rather than dysmotility-predominant pseudo-obstruction, it was not used as a primary search term; however, full texts and reference lists were reviewed for overlapping reports in which IPO was clearly described. Cases not meeting ACR criteria for SLE or lacking sufficient clinical data were excluded.

**Figure 2:**
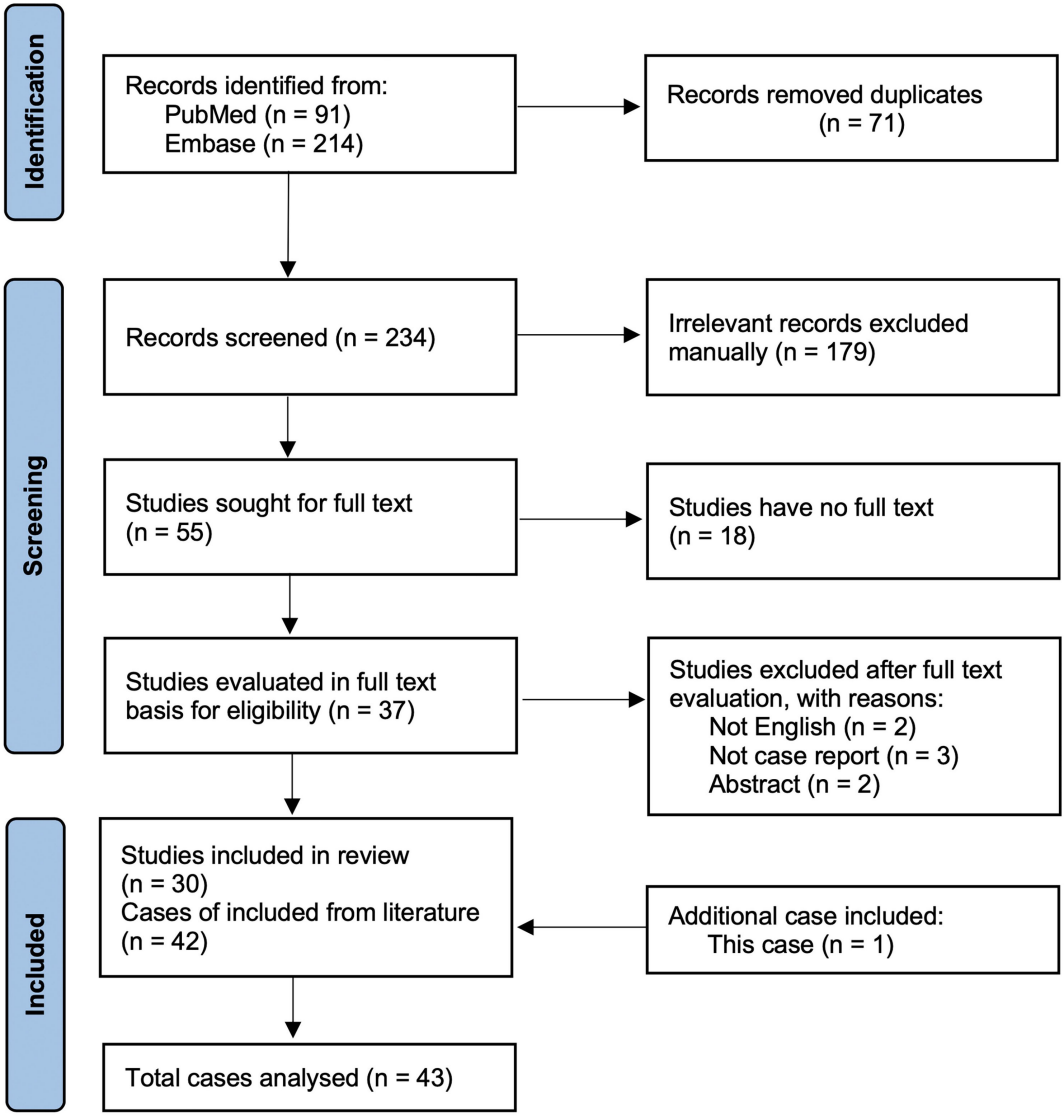
PRISMA flow diagram summarizing the selection of studies and cases included in the review. The flow chart outlines the study identification and selection process for the literature review. A total of 305 records were retrieved from PubMed (n = 91) and Embase (n = 214). After removing 71 duplicates, 234 records remained for screening. Following manual exclusion of 179 irrelevant records, 55 studies were assessed for full-text eligibility. Of these, 18 lacked full-text access and 5 were excluded because of language, non-case format, or abstract-only publication. Ultimately, 30 studies reporting 42 individual cases were included. One additional case from the present report was added, resulting in 43 cases analyzed.

The search yielded 305 records (91 from PubMed, 214 from EMBASE). After removal of 71 duplicates, 234 unique articles were screened. A total of 55 articles were selected for full-text review, and 30 articles met inclusion criteria, comprising 42 individual cases (2, 3, 6, 7, 10–34). Combined with our patient, 43 cases were included for analysis. The study selection process is summarized in a PRISMA flow diagram (**Figure 2**).

For each case, we extracted data on demographics, IPO presentation, genitourinary involvement, treatment (including corticosteroids, cyclophosphamide, IVIG, mycophenolate, rituximab), outcomes, and relapse. Cases were stratified into two groups by publication date (≤2010 vs. >2010) to explore temporal trends in treatment and prognosis. Descriptive statistics and group comparisons were performed using appropriate tests, with p<0.05 considered significant.

## Results of literature review

A total of 43 patients with SLE-associated intestinal pseudo-obstruction (IPO) were included, consisting of 42 cases from the literature and one new case (see **Table 1** for details). **Table 2** summarizes the key clinical characteristics of these patients. Female patients accounted for 93.02% (n=40), and the median age at IPO presentation was 32.00 years (range 7-51). IPO was the initial manifestation of SLE in 27 cases (62.79%). More than 90.00% of patients had active lupus at IPO onset. Genitourinary involvement—hydronephrosis, ureteral dilatation, or bladder dysfunction—was present in 20 cases (46.51%). Gastrointestinal bleeding was reported sporadically, whereas frank perforation was exceptional.

**Table 1:** Summary of 43 cases of systemic lupus erythematosus–associated intestinal pseudo-obstruction (IPO).

Source/published year(Ref.)	Published year	Sex/ age(year)	SLE duration (month)	Extra GI	Episode	Symptoms	Imaging	Active	Steroid	Other	IVIG	Surgery	Outcome
Munyard & Jaswon/ 1997 (12)	1997	F/15	0.75	Anaemia; pericardial/pleural effusion	Initial	Pain, distension, vomiting	X-ray + laparotomy	Active	Pred 60 mg/d → IV MP 0.5 g×3	HCQ	No	Exploratory laparotomy	Remission
Perlemuter/ 1998-Pt 1 (13)	1998	F/19	0	Bladder dysmotility + hydroureter	Initial	Pain, vomiting, diarrhoea, distension	Manometry + imaging	Active	IV MP pulse → pred 1 mg/kg	Cisapride	No	None	Remission
Perlemuter/ 1998-Pt 2 (13)	1998	F/28	84	Bladder + ureter	Relapse	NR	NR	Yes	High-dose steroid	No	No	None	Remission
Perlemuter/ 1998-Pt 3 (13)	1998	F/29	0	None	Initial	Pain, distension, constipation	Manometry + imaging	Yes	Steroid pulse	No	No	None	Remission
Perlemuter/ 1998-Pt 4 (13)	1998	F/39	12	Hydroureter-hydronephrosis	Relapse	NR	NR	Yes	Steroid pulse	No	No	None	Death (vasculitis)
Perlemuter/ 1998-Pt 5 (13)	1998	F/34	12	Hydroureter, bladder	Relapse	NR	NR	Yes	Steroid pulse	No	No	None	Remission
Hill/ 2000 (14)	2000	F/38	24	No	Initial	Pain, distension	X-ray, CT, path.	Yes	Pred 50mg + pulses	CsA	No	Two resections	Alive
Mok/ 2000-Pt1 (15)	2000	F/25	72	Hydroureters; ascites	Initial	Obstructive picture	CT + scope	Yes	IV MP 80 mg/d	AZA 100 mg	No	None	Two relapses, steroid-responsive
Mok/ 2000-Pt2 (15)	2000	M/25	120	Glomerulonephritis; cytopenias	Relapse	Distension, diarrhoea	X-ray + CT	Yes	IV MP 50 mg/d	AZA 100 mg	No	None	Improved
Narváez/ 2003 (16)	2003	F/44	108	No	Relapse	Pain, distension, vomiting	X-ray + SB series	Yes	MP 1 mg/kg	Cisapride; erythromycin	No	None	Lost to FU
Alexopoulou/ 2004 (3)	2004	F/32	84	Hydronephrosis	Relapse	Pain, vomiting, diarrhoea	CT	Yes	Pred 1mg/kg, daily	IV CYC	No	None	Recovered
Chen/ 2005 (17)	2005	F/47	0	Ascites	Initial	Pain, distension, vomiting	Enteroscopy + CT	Yes	Pred 1mg/kg, daily	HCQ	No	None	Recovered
Ceccato/ 2007-Pt 1 (18)	2007	F/24	12	No	Initial	Pain, distension, vomiting	X-ray, CT	Yes	Pulse MP	IV CYC	No	None	Recovered
Ceccato/ 2007-Pt 2 (18)	2007	F/21	48	Hydronephrosis, cystitis	Relapse	Pain, vomiting, weight loss	X-ray, CT, manometry	Yes	Pulse MP	IV CYC + prokinetics	No	None	Recovered
Ceccato/ 2007-Pt 3 (18)	2007	F/25	24	Hydronephrosis, cystitis	Initial	Pain, diarrhoea, fever	X-ray, CT, manometry	Yes	Pulse MP	IV CYC + prokinetics	No	None	Recovered
Ceccato/ 2007-Pt 4 (18)	2007	F/49	0	Ascites, pleural effusion	Initial	Pain, vomiting, weight loss	X-ray, CT, barium	Yes	IV MP, 1g/d×3	No	No	None	Recovered
Park/ 2009 (7)	2009	F/46	0	Hydroureter, megacholedochus	Initial	Refractory distension, vomiting, wt loss	CT + MRCP	Yes	IV MP pulse + oral	MMF; AZA	No	None	Persistent aperistalsis
Leonardi/ 2010 (19)	2010	F/51	156	Hydroureters	Relapse	Pain, obstipation	CT	No	Pred pulse	No	No	None	Improved
Kim/ 2011 (20)	2011	M/20	0	Hydroureters; colectomy	Initial	Constipation, colitis	Surgery + CT	Yes	Pred 30 mg/d ×3	AZA	No	Subtotal colectomy	Improved
Kansal/ 2012 (21)	2012	F/32	84	Hydroureteronephrosis; biliary dilation	Relapse	Severe pain, ileus	X-ray + CT	Yes	MP 0.5 g/d ×3	IV CYC	No	None	Resolved
Khairullah/ 2013 (22)	2013	F/42	0	Proteinuria	Initial	Pain, distension	CT	Yes	High-dose steroids	HCQ; AZA	No	None	Improved
García/ 2014 Pt-1 (2)	2014	F/27	0	Nephrotic, sepsis	Initial	Pain, obstruction	X-ray, CT	Yes	MP 1g/d, ×5d	IV CYC	Yes	None	Recovered
García/ 2014 Pt-2 (2)	2014	F/23	0	Sepsis, ureteritis	Initial	Pain, obstruction	X-ray, CT, manometry	Yes	MP 1g/d, ×3d	IV CYC	Yes	None	Recovered
García/ 2014 Pt-3 (2)	2014	F/25	1	HTN:KI, sepsis	Initial	Pain, vomiting	X Ray, CT	Yes	MP 1g/d, ×3d	IV CYC	No	Appendectomy prior	Alive (relapse)
García/ 2014 Pt-4 (2)	2014	F/37	144	ESRD on HD	Relapse	Diarrhoea, pain	CT	Yes	Pred 1mg/kg	No	No	None	Recurrent
Wang/ 2014 (28)	2014	F/42	0	None	Initial	Recurrent vomiting, distension, wt loss	X-ray & CT; laparotomy	Yes	MP 1 g×3 d → pred 1.5 mg/kg	No	No	Adhesiolysis (unneeded)	Remission 8 y
Oh/ 2015 (24)	2015	F/43	0	No	Initial	Nausea, vomiting, obstipation, marked distension	X-ray + CT	no	Pred 40 mg/d×7d (poor adherence)	No	No	None	Recovered
Ji/ 2016 (25)	2016	F/26	0	Acute lupus pneumonitis	Initial	Pain, vomiting, ileus	CT, path.	Yes	MP 2mg/kg/day	IVIG	Yes	Jejunal resection	Survived
Shirley/ 2017-Pt 1 (26)	2017	F/38	207	Hydroureter, neurogenic bladder	Relapse	Recurrent pain, vomiting, weight loss	X-ray & CT	Yes	Pred 1 mg/kg; later MP 500 mg	MMF	No	Exploratory laparotomy	Improved
Shirley/ 2017-Pt 2 (26)	2017	F/23	12	Bilat hydronephrosis, cystitis	Relapse	Distension, pain, loose stool	X-ray + CT	Yes	IV HC 100 mg q8h → MP 500 mg	AZA	No	None	Recovery
Álvarez-Perdomo/ 2018 (27)	2018	M/16	5	ALO, renal, pulmonary	Initial	Pain, bloating, obstipation	X-ray, CT	Yes	High-dose steroids	IVIG + MMF	Yes	None	Recovered
Wang/ 2018 (23)	2018	F/49	12	Lupus nephritis III+V	Relapse	Severe distension, vomiting	X-ray, CT	Yes	IV MP 1g, ×5d→ MP 2mg/kg/day	No	No	None	Remission
Adler/ 2019 (6)	2019	F/40	120	Bilateral hydronephrosis	Relapsing	Pain, distension, vomiting	CT	Yes	IV MP 1g, ×3d, → oral 60mg	CYC+MMF	No	None	Recovered
Nyabera/ 2020 (29)	2020	F/36	0	Hydroureters; ascites; thrombocytopenia	Initial	Vomiting, pain, distension, diarrhoea, fever	X-ray + CT	Yes	IV MP 1g, ×5d	CYC+ IVIG	Yes	None	Improved
Ayari/ 2021 (30)	2021	F/37	0	Pleural effusion, mild hydronephrosis	Initial	Pain, distension, vomiting	X-ray + CT	Yes	IV MP 1g, ×3d→1mg/kg/day	HCQ	No	None	Recovered
Hsu/ 2021 (10)	2021	F/7	0	Hepatitis, hematologic	Initial	Pain, emesis, ileus	Imaging + manometry	Yes	MP 30 mg/kg/d×3d	Rituximab + CYC	Yes	None	Recovered
Lan/ 2022 (31)	2022	F/18	1	Castleman disease; rash; lymphadenopathy	Initial	Pain, distension, vomiting	CT (air-fluid levels; no block)	Yes	Methylpred 200 mg/d → 40 mg/d	MMF	No	None	Remission
Mustafayeva/ 2022 (32)	2022	F/41	72	No	Relapse	Constipation, bloating, pain	X-ray + CT	No	Pred 1 g ×3 pulses	CYC + Rituximab	No	NG tube	Recovery
Ohri/ 2022 (11)	2022	F/12	0	Class V LN, AKI, pleural effusion	Initial	Distension, bilious vomiting, constipation, ascites	X-ray + CT	Active	IV MP pulses → oral	CYC + MMF	Yes	None	Remission
Qi/ 2022 (5)	2022	F/22	84	Persistent bilat hydroureter	Sequela	Dysuria, urgency	CT-urography	No	Pred 15 mg/d	CYC 0.4 g/wk ×6 m	No	JJ-stents	Hydronephrosis unchanged
Naeem/ 2023 (33)	2023	F/36	0	Lupus enteritis; hydronephroureter; neuro-psychosis	Initial	Pain, obstipation, distension, vomiting	X-ray + CT	Yes	Methylpred 3.5 g pulse then 0.5 mg/kg	CYC 500 mg; HCQ	No	None	Improved
Patnaik/ 2024 (34)	2024	F/32	0	Lupus enteritis, LN, invasive candidiasis	Initial	Distension, pain, obstipation, IAH	CT (wall thickening)	Yes	IV HC 200 mg/d → taper	IV CYC	No	None	Recovered; normal bowel
Present Pt	2025	F/42	180	Pulmonary arterial hypertension	Initial	Distension, pain	X-ray + CT	Yes	IV MP pulses → oral	MMF	Yes	None	Remission

This table presents a detailed summary of 43 cases of intestinal pseudo-obstruction (IPO) in patients with systemic lupus erythematosus (SLE), including 42 cases extracted from the published English-language literature and one additional case from the present study. Clinical data include patient demographics, disease duration, associated extra-gastrointestinal manifestations, IPO presentation characteristics, diagnostic findings, immunosuppressive therapies used, and clinical outcomes. Active SLE was determined based on clinical or serologic activity at the time of IPO onset. Treatments refer to acute and maintenance immunosuppressive regimens. Cases are listed in chronological order by publication year.

F/M, female/male; SLE, systemic lupus erythematosus; IPO, intestinal pseudo-obstruction; GI, gastrointestinal; CT, omputed tomography; MRI/MRCP, magnetic resonance (cholangiopancreatography); SB series, small bowel series; Pred, prednisone; MP, methylprednisolone; IV MP, intravenous methylprednisolone; CYC, cyclophosphamide; AZA, azathioprine; MMF, mycophenolate mofetil; RTX, rituximab; HCQ, hydroxychloroquine; IVIG, intravenous immunoglobulin; ALO, acute lupus onset; LN, lupus nephritis; ARF, acute renal failure; V.N, visceral neuropathy; L.N, lymphadenopathy; JJ-stent, double J ureteral stent; NG tube, nasogastric tube; NR, not reported; Pt, patient; →, followed by.

**Table 2:** Summary of clinical characteristics, imaging findings, treatments, and outcomes in 43 patients with SLE-associated intestinal pseudo-obstruction (IPO).

Characteristic	Value (N = 43 cases)
Female: Male ratio	40:3 (93.02% female)
Age at IPO presentation	Median 32 years (range 7–51)
IPO as initial SLE manifestation	27 cases (62.79%)
SLE duration in others	Median 24 months (range 3–180) in 16 known-SLE cases
Active SLE at IPO onset	>90% (almost all cases had active disease signs/serologies)
Extra-intestinal involvement:	
– Genitourinary (hydronephrosis, etc)	20 cases (46.51%)
– Serositis (pleural/pericardial)	9 cases (20.93%)
– Lupus enteritis (mesenteric vas.)	8 cases (18.60%) (overlap suspected or confirmed)
– Lupus nephritis	5 cases (11.63%) (notably Class IV in a few)
– Pulmonary arterial hypertension	1 case (our patient)
Imaging findings:	All with dilated bowel + air-fluid levels; 20% with biliary dilation; ~50% with ureteric dilation
Endoscopic biopsy results:	Usually normal or nonspecific; full-thickness biopsy (few cases) showed visceral myositis, minimal vasculitis
Treatment received:	
– Corticosteroids (high-dose)	43 (100.00%)
– Cyclophosphamide (IV or oral)	16 (37.21%)
– Mycophenolate mofetil	7 (16.28%)
– IV immunoglobulin	8 (18.60%)
– Rituximab	2 (4.65%)
– Exploratory surgery (due to misdx)	11 (25.58%) – often non-therapeutic, prior to IPO diagnosis
Outcome:	
– Complete resolution	27 (62.79%)
– Partial improvement (residual)	15 (34.88%)
– Death	1 (2.33%)
Relapse of IPO	17 (39.53%) (typically managed with repeat immunosuppression)

This table summarizes aggregated data from 43 reported cases of intestinal pseudo-obstruction (IPO) in systemic lupus erythematosus (SLE), including demographic features, IPO onset patterns, SLE activity, extra-intestinal manifestations, imaging and biopsy findings, treatment modalities, clinical outcomes, and recurrence rates. Frequencies are presented as absolute values and percentages. IPO was defined by radiologic evidence of bowel dilation without mechanical obstruction. Active SLE was assessed by serologic or clinical disease activity at the time of IPO onset. Genitourinary involvement includes hydronephrosis, ureteral dilatation, or bladder dysfunction.

SLE, systemic lupus erythematosus; IPO, intestinal pseudo-obstruction; GI, gastrointestinal; IV, intravenous; CT, computed tomography; PAH, pulmonary arterial hypertension; IVIG, intravenous immunoglobulin; MMF, mycophenolate mofetil.

All patients were treated with high-dose corticosteroids. Cyclophosphamide (CYC) was used in 16 cases (37.21%), intravenous immunoglobulin (IVIG) in 8 cases (18.60%), mycophenolate mofetil (MMF) in 7 cases (16.28%), and rituximab (RTX) in 2 cases (4.65%). Exploratory surgery was performed in 11 cases (25.58%), usually before the diagnosis of IPO was established. IPO relapsed in 17 patients (39.53%), most of whom responded to retreatment. Complete resolution was reported in 27 patients (62.79%), partial improvement in 15 (34.88%), and death in 1 (2.33%) (13).

To assess temporal trends, patients were divided into two groups by publication year: ≤2010 (n=18) and >2010 (n=25). Key comparisons are shown in **Table 3**.

**Table 3:** Summary of clinical characteristics, treatment strategies, and outcomes in SLE‑IPO cases reported before and after 2010.

Variable	2010 and Earlier (n=18)	2011 and Later (n=25)	*p*-value
Female: Male ratio	17:1 (94.44% F)	23:2 (92.00% F)	1.00
Age at IPO onset (years)	32.83 ± 11.13 (median 30.50)	30.64 ± 11.03 (median 32.00)	0.53
IPO as initial SLE manifestation	10 (55.56%)	17 (68.00%)	0.53
Urinary tract involvement	10 (55.56%)	10 (40.00%)	0.47
Steroid used	18 (100.00%)	25 (100.00%)	–
Cyclophosphamide use	4 (22.22%)	12 (48.00%)	0.12
Mycophenolate use	1 (5.56%)	6 (24.00%)	0.21
IVIG use	0 (0.00%)	8 (32.00%)	0.01
Rituximab use	0 (0.00%)	2 (8.00%)	0.50
IPO relapse rate	9 (50.00%)	8 (32.00%)	0.34
Outcome – complete remission	11 (61.11%)	16 (64.00%)	0.49¹
Outcome – partial improvement	6 (33.33%)	9 (36.00%)	0.49¹
Outcome – mortality	1 (5.56%)	0 (0.00%)	–

This table shows clinical characteristics, treatment strategies, and outcomes in SLE-IPO cases reported before and after 2010. Percentages reflect the proportion within each group. IVIG = intravenous immunoglobulin; CYC = cyclophosphamide; MMF = mycophenolate mofetil; RTX = rituximab. Age is presented as mean ± standard deviation (median). Relapse was inferred from case labels or clinical course when specified.

While demographic characteristics were broadly similar, the >2010 group more frequently received IVIG, MMF, and RTX, with IVIG use increasing from 0.00% to 32.00%. IPO relapse appeared less common in recent reports (32.00% vs. 50.00%), and no deaths were reported in the >2010 group. These temporal differences may reflect improved recognition and evolving treatment strategies, but they should be interpreted cautiously because of the small subgroup sizes and heterogeneous case reporting.

## Discussion

This case-based review provides an updated synthesis of the clinical characteristics, management, and outcomes of SLE-associated intestinal pseudo-obstruction (SLE-IPO), incorporating 42 previously published cases plus our own. The findings reinforce key clinical messages while highlighting evolving treatment practices over time.

SLE-IPO typically affects young female patients and can be the initial manifestation of lupus (4, 24, 29, 30), as seen in over 60% of cases in our review. Despite presenting with signs suggestive of mechanical bowel obstruction, IPO lacks an anatomical cause and is frequently misdiagnosed, leading to delayed treatment and unnecessary surgery. Our case illustrates this diagnostic challenge and underscores the importance of multidisciplinary involvement to recognize IPO early.

One of the most striking associations in SLE-IPO is genitourinary involvement, seen in nearly half of patients. This supports the hypothesis that lupus-related visceral smooth-muscle dysfunction underlies both gastrointestinal and urinary manifestations. Other frequent extra-gastrointestinal findings include serositis and hematologic abnormalities (10, 12, 15).

Rarely, IPO may coincide with pulmonary arterial hypertension (PAH), as observed in our case. This association has been reported only once before in the literature (35) and may reflect a shared pathogenic mechanism involving lupus-related microvascular injury or smooth-muscle dysfunction affecting both pulmonary arterioles and visceral musculature. In our patient, PAH was diagnosed early in the disease course and improved after immunosuppressive therapy, whereas IPO occurred 15 years later during a disease flare. APS-related microvascular disease was considered because lupus anticoagulant was positive at relapse; however, anticardiolipin and anti-β2-glycoprotein I antibodies were negative, no macrovascular thrombosis or mesenteric ischemia was identified on contrast-enhanced CT, and the patient improved without escalation of anticoagulation. A thrombotic or small-vessel vasculitic component therefore cannot be completely excluded, particularly in the absence of full-thickness histology, but the overall presentation favored inflammatory SLE-related visceral dysmotility. Anti-RNP positivity, Raynaud’s phenomenon, PAH, and gastrointestinal dysmotility may also raise the possibility of an overlap connective tissue disease such as systemic sclerosis or mixed connective tissue disease. Nevertheless, the patient had longstanding dsDNA-positive SLE, no interstitial lung disease on chest CT, and no documented defining scleroderma features in the available records, making active SLE the most parsimonious explanation.

Therapeutically, all patients received corticosteroids, but adjunct immunosuppressive use varied. Our analysis suggests increased use of intravenous immunoglobulin (IVIG) (11, 29), mycophenolate mofetil (MMF) (6, 26), and rituximab (RTX) (10, 32) in cases reported after 2010, reflecting a shift toward more individualized and escalated immunosuppressive strategies. Notably, IVIG use rose from 0.00% to 32.00% between eras, likely because of its immunomodulatory role in severe or steroid-refractory disease. MMF is a valuable maintenance agent, but in our case, it was introduced only after bowel function had recovered and oral absorption was considered reliable; we do not suggest MMF as initial rescue therapy during the obstructive phase.

An additional interpretive issue is the relationship between IPO and lupus enteritis. We intentionally focused our primary search on dysmotility-related terms because “lupus enteritis” is more often used for vasculitic or inflammatory bowel-wall edema than for a motility-dominant syndrome. Nonetheless, some published cases showed overlapping features, suggesting that pseudo-obstruction and lupus enteritis may coexist along a spectrum of gastrointestinal lupus involvement. In the literature we reviewed, gastrointestinal bleeding was uncommon and frank perforation was rare, but both underscore the need for close monitoring in severe disease.

Relapse occurred in a substantial minority of cases, but most patients were responsive to retreatment. Only one death was reported in the entire cohort (13), and none in the last decade, underscoring the generally favorable prognosis with prompt and appropriate therapy. However, persistent or relapsing motility impairment remains a concern and warrants long-term follow-up. This study is limited by the nature of available evidence—primarily case reports and small case series—with substantial heterogeneity in diagnostic work-up, treatment regimens, follow-up duration, and outcome definitions. Publication bias is also likely, because unusual presentations and successful therapeutic responses are more likely to be reported than negative or unremarkable cases. In addition, several subgroup comparisons were based on small numbers and should be interpreted as exploratory rather than confirmatory. Accordingly, our pooled analysis can identify clinical patterns and temporal practice changes, but it cannot establish treatment efficacy or causal relationships.

## Conclusion

SLE-associated intestinal pseudo-obstruction is an uncommon but treatable manifestation of lupus, often presenting with acute abdominal symptoms and functional urinary tract involvement. Prompt recognition and immunosuppressive therapy, especially corticosteroids, are critical to avoid irreversible bowel damage and surgical interventions. Over the past decade, increased use of IVIG, MMF, and biologics reflects evolving treatment strategies. Most patients recover fully or partially, and mortality is now rare. Clinicians should maintain a high index of suspicion for IPO in lupus patients with unexplained ileus or visceral dilatation and initiate timely, aggressive therapy tailored to disease severity. Because current evidence is case-based and heterogeneous, treatment-outcome associations should be interpreted cautiously.

## Data Availability

The original contributions presented in the study are included in the article/supplementary material. Further inquiries can be directed to the corresponding author.

## References

[1] Patricio JPN Ngo JD Navarra SV . Gastrointestinal involvement in systemic lupus erythematosus. Int J Rheumatic Dis. (2014) 17:69. Conference Abstract.

[2] García López CA Laredo-Sánchez F Malagón-Rangel J Flores-Padilla MG Nellen-Hummel H . Intestinal pseudo-obstruction in patients with systemic lupus erythematosus: a real diagnostic challenge. World J Gastroenterol. (2014) 20:11443–50. doi:10.3748/wjg.v20.i32.11443. PMID: 25170234 PMC4145788

[3] Alexopoulou A Andrianakos A Dourakis SP . Intestinal pseudo-obstruction and ureterohydronephrosis as the presenting manifestations of relapse in a lupus patient. Lupus. (2004) 13:954–6. doi:10.1191/0961203304u1064cr. PMID: . Article. 15645752

[4] Jin P Ji X Zhi H Song X Du H Zhang K . A review of 42 cases of intestinal pseudo-obstruction in patients with systemic lupus erythematosus based on case reports. Hum Immunol. (2015) 76:695–700. doi:10.1016/j.humimm.2015.09.022. PMID: 26429329

[5] Qi W Zhou Y Zhao J . Bilateral ureterohydronephrosis after intestinal pseudo-obstruction in a patient with systemic lupus erythematosus. Rheumatol Immunol Res. (2022) 3:211–2. doi:10.2478/rir-2022-0037. PMID: 36879835 PMC9984930

[6] Adler BL Timlin H Birnbaum J . Lupus intestinal pseudo-obstruction and hydronephrosis: Case report. Med (Baltimore). (2019) 98:e16178. doi:10.1097/md.0000000000016178. PMID: 31305400 PMC6641825

[7] Park FD Lee JK Madduri GD Ghosh P . Generalized megaviscera of lupus: refractory intestinal pseudo-obstruction, ureterohydronephrosis and megacholedochus. World J Gastroenterol. (2009) 15:3555–9. doi:10.3748/wjg.15.3555. PMID: 19630114 PMC2715985

[8] Huang Q Lai W Yuan C Shen S Cui D Zhao J . Predictors of intestinal pseudo-obstruction in systemic lupus erythematosus complicated by digestive manifestations: data from a Southern China lupus cohort. Lupus. (2016) 25:248–54. doi:10.1177/0961203315605366. PMID: 26405024

[9] Zhang L Xu D Yang H Tian X Wang Q Hou Y . Clinical features, morbidity, and risk factors of intestinal pseudo-obstruction in systemic lupus erythematosus: A retrospective case-control study. J Rheumatol. (2016) 43:559–64. doi:10.3899/jrheum.150074. PMID: . Article. 26773109

[10] Hsu D Khalsa UK Hassan M Sandborg CI Namjoshi SS . Early intervention and resolution of pediatric intestinal pseudo-obstruction in systemic lupus erythematosus: A pediatric case report. JPGN Rep. (2021) 2:e041. doi:10.1097/pg9.0000000000000041. PMID: 37206925 PMC10191485

[11] Ohri AJ Shah CG Udani AH . Intestinal pseudo-obstruction - An under-recognized presentation of systemic lupus erythematosus. Indian J Nephrol. (2022) 32:476–9. doi:10.4103/ijn.IJN_494_20. PMID: 36568587 PMC9775609

[12] Munyard P Jaswon M . Systemic lupus erythematosus presenting as intestinal pseudo-obstruction. J R Soc Med. (1997) 90:48–9. doi:10.1177/014107689709000117. PMID: 9059386 PMC1296120

[13] Perlemuter G Chaussade S Wechsler B Cacoub P Dapoigny M Kahan A . Chronic intestinal pseudo-obstruction in systemic lupus erythematosus. Gut. (1998) 43:117–22. doi:10.1136/gut.43.1.117. PMID: 9771415 PMC1727185

[14] Hill PA Dwyer KM Power DA . Chronic intestinal pseudo-obstruction in systemic lupus erythematosus due to intestinal smooth muscle myopathy. Lupus. (2000) 9:458–63. doi:10.1191/096120300678828505. PMID: . Article. 10981652

[15] Mok MY Wong RWS Lau CS . Intestinal pseudo-obstruction in systemic lupus erythematosus: An uncommon but important clinical manifestation. Lupus. (2000) 9:11–8. doi:10.1177/096120330000900104. PMID: . Article. 10713642

[16] Narváez J Pérez-Vega C Castro-Bohorquez FJ Garcia-Quintana AM Biosca M Vilaseca-Momplet J . Intestinal pseudo-obstruction in systemic lupus erythematosus. Scandinavian J Rheumatol. (2003) 32:191–5. doi:10.1080/03009740310002588. PMID: . Article. 12892261

[17] Chen YY Yen HH Hsu YT . Intestinal pseudo-obstruction as the initial presentation of systemic lupus erythematosus: The need for enteroscopic evaluation. Gastrointestinal Endoscopy. (2005) 62:984–7. doi:10.1016/j.gie.2005.08.010. PMID: . Article. 16301053

[18] Ceccato F Salas A Góngora V Ruta S Roverano S Marcos JC . Chronic intestinal pseudo-obstruction in patients with systemic lupus erythematosus: report of four cases. Clin Rheumatol. (2008) 27:399–402. doi:10.1007/s10067-007-0760-5. PMID: 17938989

[19] Leonardi G de Bortoli N Bellini M Mumolo MG Costa F Ricchiuti A . Intestinal pseudo-obstruction in inactive systemic lupus erythematosus: An unusual finding. World J Gastrointest Pharmacol Ther. (2010) 1:135–6. doi:10.4292/wjgpt.v1.i6.135. PMID: 21577309 PMC3091158

[20] Kim J Kim N . Intestinal pseudo-obstruction: initial manifestation of systemic lupus erythematosus. J Neurogastroenterol Motil. (2011) 17:423–4. doi:10.5056/jnm.2011.17.4.423. PMID: 22148114 PMC3228985

[21] Kansal A Jain A Thenozhi S Agarwal V . Intestinal pseudo-obstruction associated with biliary tract dilatation in a patient with systemic lupus erythematosus. Lupus. (2013) 22:87–91. doi:10.1177/0961203312464091. PMID: 23104391

[22] Khairullah S Jasmin R Yahya F Cheah TE Ng CT Sockalingam S . Chronic intestinal pseudo-obstruction: a rare first manifestation of systemic lupus erythematosus. Lupus. (2013) 22:957–60. doi:10.1177/0961203313492873. PMID: 23761180

[23] Wang R Zheng B Wang B Ma P Chen F Tang L . A report of chronic intestinal pseudo-obstruction related to systemic lupus erythematosus. Open Med (Wars). (2018) 13:562–4. doi:10.1515/med-2018-0083. PMID: 30519633 PMC6272055

[24] Oh DJ Yang JN Lim YJ Kang JH Park JH Kim MY . Intestinal pseudo-obstruction as an initial manifestation of systemic lupus erythematosus. Intest Res. (2015) 13:282–6. doi:10.5217/ir.2015.13.3.282. PMID: 26131004 PMC4479744

[25] Ji C Yu X Wang Y Shi L . Acute lupus pneumonitis followed by intestinal pseudo-obstruction in systemic lupus erythematosus: A case report. Exp Ther Med. (2016) 12:245–9. doi:10.3892/etm.2016.3316. PMID: 27347044 PMC4906679

[26] Shirley L Thundyil RJ . A frequently missed entity in systemic lupus erythematosus (SLE): Intestinal pseudo-obstruction (IpsO). Med J Malaysia. (2017) 72:374–5. doi:10.1007/978-0-306-48113-0_426. PMID: 29308779

[27] Álvarez-Perdomo LC Pedraza-Atahualpa PA Mondragón-Cardona ÁE Escobar-Montealegre F Jiménez-Canizales CE . An unusual case of intestinal pseudo-obstruction presenting in an adolescent with juvenile-onset systemic lupus erythematosus: A diagnostic challenge. Egyptian Rheumatologist. (2018) 40:213–6. doi:10.1016/j.ejr.2017.10.003. PMID: . Article. 41936479

[28] Wang JL Liu G Liu T Wei JP . Intestinal pseudo-obstruction in systemic lupus erythematosus: a case report and review of the literature. Med (Baltimore). (2014) 93:e248. doi:10.1097/md.0000000000000248. PMID: 25546663 PMC4602607

[29] Nyabera A Elfishawi M Cuevas F Riaz F Abrudescu A . Intestinal pseudo-obstruction as the initial clinical presentation in systemic lupus erythematosus: A rare and severe disorder. Case Rep Gastrointest Med. (2020) 2020:8873917. doi:10.1155/2020/8873917. PMID: 33274088 PMC7683163

[30] Ayari M Nakhli A Teyeb Z Abdelaali I Bellakhal S Jomni T . Intestinal pseudo-obstruction: Unusual presentation of systemic lupus erythematous. Clin Case Rep. (2021) 9:1759–62. doi:10.1002/ccr3.3907. PMID: 33768930 PMC7981693

[31] Lan F Li T Zhou L Long L Liu L Li X . Intestinal pseudo-obstruction in systemic lupus erythematosus complicated by Castleman disease: a case report. Ann Transl Med. (2022) 10:1148. doi:10.21037/atm-22-4461. PMID: 36388834 PMC9652566

[32] Mustafayeva L Durucan I Ayla AY Ugurlu S . Systemic lupus erythematosus-related intestinal pseudo-obstruction treated with immunosuppressants. Lupus Sci Med. (2022) 9:A62. doi:10.1136/lupus-2022-elm2022.97. PMID: . Conference Abstract. 41926762

[33] Naeem F Noor MU Batool S Anwer Khan SE Akmal M . An atypical initial manifestation of systemic lupus erythematosus: Lupus enteritis accompanied by intestinal pseudo-obstruction and bilateral hydronephroureter. Cureus. (2023) 15:e50628. doi:10.7759/cureus.50628. PMID: 38226118 PMC10789390

[34] Patnaik R Chawla A Ramakrishnan AB Jain N Molazadeh N Pillai A . An uncommon manifestation of systemic lupus erythematosus as lupus enteritis with intestinal pseudo-obstruction and invasive candidiasis. Cureus. (2024) 16:e70485. doi:10.7759/cureus.70485. PMID: 39479075 PMC11522948

[35] Gilyarov MY Shostak NA Kotovа DP Schekochikhin DY Kasha YO . Clinical observation pseudoobstruction syndrome of the stomach's output part and small intestine of a patient with systematic lupus erythematosis. Ter Arkh. (2018) 90:98–101. doi:10.26442/terarkh201890298-101. PMID: 30701783

